# Supplementary material for: Multi‐dataset identification of innovative feature genes and molecular mechanisms in keratoconus
Source: J Cell Mol Med. 2024 Sep 19;28(18):e70079. doi: 10.1111/jcmm.70079 (PMC11412914; doi:10.1111/jcmm.70079)
Supplement: Supplementary file 2 — Material S2. [file JCMM-28-e70079-s002.zip › 7.GSEA analysis/5.RNF217-AS1/KEGG_RNA_DEGRADATION.html]

Details for gene set KEGG\_RNA\_DEGRADATION[GSEA]

|  || Dataset | RNF217-AS1.group.cls#h\_versus\_l |
| Phenotype | group.cls#h\_versus\_l |
| Upregulated in class | h |
| GeneSet | KEGG\_RNA\_DEGRADATION |
| Enrichment Score (ES) | 0.2233981 |
| Normalized Enrichment Score (NES) | 1.1518955 |
| Nominal p-value | 0.24621212 |
| FDR q-value | 1.0 |
| FWER p-Value | 0.993 |
Table: GSEA Results Summary

  

Fig 1: Enrichment plot: KEGG\_RNA\_DEGRADATION      
 Profile of the Running ES Score & Positions of GeneSet Members on the Rank Ordered List

  

| SYMBOL | TITLE | RANK IN GENE LIST | RANK METRIC SCORE | RUNNING ES | CORE ENRICHMENT || 1 | ENO2 | na | 4779 | 0.225 | -0.0466 | Yes |
| 2 | CNOT1 | na | 7083 | 0.150 | -0.0519 | Yes |
| 3 | PATL1 | na | 8667 | 0.113 | -0.0519 | Yes |
| 4 | CNOT2 | na | 10954 | 0.071 | -0.0891 | Yes |
| 5 | DIS3 | na | 11145 | 0.068 | -0.0670 | Yes |
| 6 | PAPOLB | na | 11295 | 0.066 | -0.0445 | Yes |
| 7 | PAPOLA | na | 11352 | 0.065 | -0.0197 | Yes |
| 8 | EXOSC6 | na | 12024 | 0.055 | -0.0167 | Yes |
| 9 | EXOSC3 | na | 13653 | 0.034 | -0.0497 | Yes |
| 10 | PAPOLG | na | 14319 | 0.027 | -0.0580 | Yes |
| 11 | DDX6 | na | 14631 | 0.023 | -0.0575 | Yes |
| 12 | DCP1A | na | 15638 | 0.013 | -0.0815 | Yes |
| 13 | CNOT3 | na | 15792 | 0.011 | -0.0815 | Yes |
| 14 | DCP2 | na | 15891 | 0.010 | -0.0803 | Yes |
| 15 | EXOSC4 | na | 15948 | 0.009 | -0.0782 | Yes |
| 16 | DCPS | na | 16909 | -0.001 | -0.1056 | Yes |
| 17 | EXOSC9 | na | 17046 | -0.002 | -0.1087 | Yes |
| 18 | EXOSC1 | na | 17059 | -0.002 | -0.1081 | Yes |
| 19 | LSM7 | na | 17602 | -0.007 | -0.1208 | Yes |
| 20 | C1D | na | 17782 | -0.009 | -0.1224 | Yes |
| 21 | CNOT6 | na | 17804 | -0.009 | -0.1194 | Yes |
| 22 | ENO1 | na | 18144 | -0.012 | -0.1243 | Yes |
| 23 | CNOT6L | na | 18215 | -0.013 | -0.1211 | Yes |
| 24 | CNOT10 | na | 18863 | -0.019 | -0.1321 | Yes |
| 25 | ENO3 | na | 18968 | -0.020 | -0.1271 | Yes |
| 26 | PARN | na | 19356 | -0.024 | -0.1286 | Yes |
| 27 | LSM8 | na | 19360 | -0.024 | -0.1190 | Yes |
| 28 | EXOSC8 | na | 19454 | -0.025 | -0.1115 | Yes |
| 29 | LSM5 | na | 19661 | -0.027 | -0.1066 | Yes |
| 30 | EXOSC2 | na | 19810 | -0.028 | -0.0995 | Yes |
| 31 | CNOT7 | na | 20056 | -0.031 | -0.0941 | Yes |
| 32 | XRN2 | na | 20067 | -0.031 | -0.0819 | Yes |
| 33 | EXOSC10 | na | 20276 | -0.033 | -0.0747 | Yes |
| 34 | HSPD1 | na | 21105 | -0.041 | -0.0819 | Yes |
| 35 | LSM1 | na | 21191 | -0.042 | -0.0673 | Yes |
| 36 | LSM3 | na | 21806 | -0.049 | -0.0653 | Yes |
| 37 | EXOSC7 | na | 21842 | -0.049 | -0.0464 | Yes |
| 38 | EDC3 | na | 21976 | -0.051 | -0.0297 | Yes |
| 39 | EDC4 | na | 22050 | -0.051 | -0.0110 | Yes |
| 40 | LSM6 | na | 22187 | -0.053 | 0.0065 | Yes |
| 41 | XRN1 | na | 23089 | -0.063 | 0.0060 | Yes |
| 42 | HSPA9 | na | 23181 | -0.064 | 0.0293 | Yes |
| 43 | LSM2 | na | 23308 | -0.066 | 0.0523 | Yes |
| 44 | PNPT1 | na | 23526 | -0.069 | 0.0739 | Yes |
| 45 | LSM4 | na | 23576 | -0.069 | 0.1007 | Yes |
| 46 | CNOT4 | na | 23981 | -0.075 | 0.1194 | Yes |
| 47 | ZCCHC7 | na | 24019 | -0.076 | 0.1490 | Yes |
| 48 | CNOT8 | na | 24258 | -0.079 | 0.1742 | Yes |
| 49 | EXOSC5 | na | 24587 | -0.084 | 0.1988 | Yes |
| 50 | DCP1B | na | 25905 | -0.105 | 0.2036 | Yes |
| 51 | MPHOSPH6 | na | 26981 | -0.125 | 0.2234 | Yes |
Table: GSEA details [plain text format]

  

Fig 2: KEGG\_RNA\_DEGRADATION      
 Blue-Pink O' Gram in the Space of the Analyzed GeneSet

  

Fig 3: KEGG\_RNA\_DEGRADATION: Random ES distribution      
 Gene set null distribution of ES for **KEGG\_RNA\_DEGRADATION**
